# Malocclusion impairs cognitive behavior via AgRP signaling in adolescent mice

**DOI:** 10.3389/fnins.2023.1156523

**Published:** 2023-04-24

**Authors:** Junya Kusumoto, Koji Ataka, Haruki Iwai, Yasuhiko Oga, Keita Yamagata, Kanako Marutani, Takanori Ishikawa, Akihiro Asakawa, Shouichi Miyawaki

**Affiliations:** ^1^Department of Orthodontics and Dentofacial Orthopedics, Field of Developmental Medicine, Health Research Course, Kagoshima University Graduate School of Medical and Dental Sciences, Kagoshima, Japan; ^2^Laboratory of Medical Biochemistry, Kobe Pharmaceutical University, Kobe, Japan; ^3^Department of Psychosomatic Internal Medicine, Kagoshima University Graduate School of Medical and Dental Sciences, Kagoshima, Japan; ^4^Department of Oral Anatomy and Cell Biology, Kagoshima University Graduate School of Medical and Dental Sciences, Kagoshima, Japan; ^5^Department of Orthodontics, Center of Developmental Dentistry, Kagoshima University Hospital, Kagoshima, Japan; ^6^Department of Orthodontics, Dentistry and Pharmaceutical Sciences, Okayama University Graduate School of Medicine, Okayama, Japan

**Keywords:** adolescent, agouti-related protein, arcuate nucleus of hypothalamus, cognitive dysfunction, malocclusion, novel object recognition test

## Abstract

**Introduction:**

Occlusal disharmony induced by deteriorating oral health conditions, such as tooth loss and decreased masticatory muscle due to sarcopenia, is one of the causes of cognitive impairment. Chewing is an essential oral function for maintaining cognitive function not only in the elderly but also in young people. Malocclusion is an occlusal disharmony that commonly occurs in children. The connection between a decline in cognitive function and malocclusion in children has been shown with chronic mouth breathing, obstructive sleep apnea syndrome, and thumb/digit sucking habits. However, the mechanism of malocclusion-induced cognitive decline is not fully understood. We recently reported an association between feeding-related neuropeptides and cognitive decline in adolescent mice with activity-based anorexia. The aim of the present study was to assess the effects of malocclusion on cognitive behavior and clarify the connection between cognitive decline and hypothalamic feeding-related neuropeptides in adolescent mice with malocclusion.

**Methods:**

Four-week-old mice were randomly assigned to the sham-operated solid diet-fed (Sham/solid), sham-operated powder diet-fed (Sham/powder), or malocclusion-operated powder diet-fed (Malocclusion/powder) group. We applied composite resin to the mandibular anterior teeth to simulate malocclusion. We evaluated cognitive behavior using a novel object recognition (NOR) test, measured hypothalamic feeding-related neuropeptide mRNA expression levels, and enumerated c-Fos-positive cells in the hypothalamus 1 month after surgery. We also evaluated the effects of central antibody administration on cognitive behavior impairment in the NOR test.

**Results:**

The NOR indices were lower and the agouti-related peptide (AgRP) mRNA levels and number of c-Fos-positive cells were higher in the malocclusion/powder group than in the other groups. The c-Fos-positive cells were also AgRP-positive. We observed that the central administration of anti-AgRP antibody significantly increased the NOR indices.

**Discussion:**

The present study suggests that elevated cerebral AgRP signaling contributes to malocclusion-induced cognitive decline in adolescents, and the suppression of AgRP signaling can be a new therapeutic target against cognitive decline in occlusal disharmony.

## 1. Introduction

Mastication is a sensory-motor function wherein food is chewed, ground, and swallowed (López-Chaichio et al., 2021). Masticatory dysfunction, such as malocclusion, leads to abnormal sensory input and is associated with cognitive impairment in the elderly (Grayson et al., 2015; Weijenberg et al., 2019). Moreover, the relationship between malocclusion and cognitive impairment has become evident in the young and middle-aged. Chewing gum improves cognitive functions, such as word recall and duration of spatial working memory, in the young and middle-aged (Wilkinson et al., 2002; Baker et al., 2004; Stephens and Tunney, 2004). Malocclusion is a common dental disease in children (Lombardo et al., 2020). Children with obstructive sleep apnea-hypopnea syndrome have a higher incidence rate of malocclusion and lower score of cognitive function than the healthy control (Cai et al., 2013); those with chronic mouth breathing develop malocclusion that changes the shape of their face and mouth, and their cognitive and academic abilities decline as compared to the nasal breathing control group (Kuroishi et al., 2015; Grippaudo et al., 2016). Thumb/digit sucking habits raise a child’s risk of increased overjet and open bite followed by malocclusion and speech and language delay (Ling et al., 2018; Kumar et al., 2022). In this way, malocclusion-induced cognitive dysfunction is considered to be a serious health hazard not only to the elderly but also the young.

Children and adolescents with malocclusion experience substantial psychological discomfort and a negative impact on their oral health-related quality of life (Chen et al., 2015; Bittencourt et al., 2017). Young subjects with malocclusions present with poor responsiveness to the pain-relieving effects of relaxation (Ruscheweyh et al., 2015). Orthodontic patients who require surgical treatment display high levels of anxiety and depression (Hino et al., 2022). Thus, malocclusion is associated with stress, and chronic stress alters cognitive function (Lupien et al., 2009). Young adults subjected to psychosocial stress for 4 weeks exhibit reduced attentional control (Liston et al., 2009). Cumulative life stress, assessed by the Youth Life Stress Interview, reduces executive functions, which are high-level cognitive abilities, such as spatial working memory and cognitive flexibility, in children (Hanson et al., 2012). Repeated restraint stress for 7 days impairs cognitive function in novel object recognition (NOR) tests assigned to animals (Yuen et al., 2012).

Recently, an association between eating disorders and occlusal disharmony was revealed (Chiba et al., 2022). Anorexia nervosa is a serious eating disorder in adolescent women and causes cognitive decline, such as body image distortion (Dalhoff et al., 2019). Adolescent mice with anorexia nervosa display cognitive decline, and central inhibition of agouti-related peptide (AgRP) and neuropeptide Y (NPY) reverses the cognitive decline (Rokot et al., 2021). Thus, cerebral orexigenic peptides may alter cognitive functions in adolescents. Occlusal disharmony impairs cognitive function by upregulating hippocampal cognitive inhibitors in adult mice (Maeshiba et al., 2022). However, to the best of our knowledge, no prior study has investigated the relationship between orexigenic peptides associated with cognitive function and occlusal disharmony in adolescent mice.

The NOR test investigates cognitive paradigms based on working memory, attention, anxiety, and novelty preference in rodents lacking reward or punishment (Antunes and Biala, 2012). Rodents approach and explore novel objects frequently when they are simultaneously exposed to familiar and novel objects. The NOR test has been used to assess cognitive function in various animal models of Alzheimer’s disease, traumatic brain injury, schizophrenia, Parkinson’s disease, autism spectrum disorder, and aging (Grayson et al., 2015).

The aim of this study was to evaluate the relationship between hypothalamic orexigenic peptides and cognitive behavior deficiency in young mice with occlusal disharmony using the NOR test This study is expected to reveal the central mechanism underlying malocclusion-induced cognitive decline.

## 2. Materials and methods

### 2.1. Animals

Male C57BL/6J mice (age, 3 weeks; body weight range, 8–12 g) were purchased from Charles River Laboratories Japan Inc. (Tokyo, Japan). They were individually housed in cages at 24 ± 2°C and 50 ± 10% humidity under a 12/12 h light/dark cycle. The light period was between 07:00 and 19:00. The mice had *ad libitum* access to a sterile standard diet (3.4 kcal/g; CE-2; CLEA Japan Inc. Tokyo, Japan) and water in a pathogen-free facility. All experimental protocols were approved by the Kagoshima University Committee for Animal Experiments (No. D21035). The present study conformed to Animal Research: Reporting *In Vivo* Experiments (ARRIVE) Guidelines v.2.0 for Preclinical Animal Studies. Sample size and inclusion and exclusion criteria are described in the Supplementary methods.

### 2.2. Mouse occlusal disharmony model induction

**FIGURE 1:**
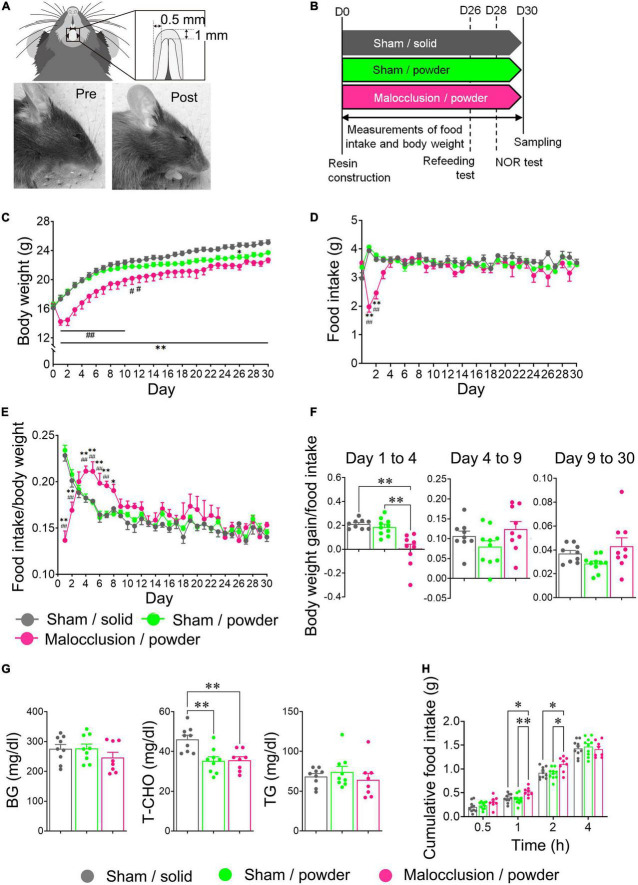
Photographs and schematic diagram of occlusal disharmony in mice, schedule diagram, and body weight and food intake time courses. **(A)** Representative image of malocclusion (1 mm increase in vertical height) induced by cementing composite resin onto mandibular incisors of mice. **(B)** Mice were assigned to Sham operation with solid chow (Sham/solid, *n* = 9), Sham operation with powder chow (Sham/powder, *n* = 10), or application of composite resin on mandibular anterior teeth with powder chow (Malocclusion/powder, *n* = 9) group. Experiments were performed according to the schedule shown. Body weight **(C)** and food intake (**D**) were measured for 30 d under experimental conditions. Food intake/body weight **(E)** and body weight gain/food intake from Day 1 to 4, Day 4 to 9, and Day 9 to 30 **(F)** were calculated. **(G)** Blood glucose (BG), total cholesterol (T-CHO), and triglyceride (TG) concentrations in peripheral blood were measured. **(H)** Cumulative food intake was measured in other mice: Sham/solid (*n* = 10), Sham/powder (*n* = 10) and Malocclusion/powder group (*n* = 8) after 16 h fasting. Data are represented as means ± SEM. In panels **(C–E)** differences were considered significant at *p < 0.05 and **p < 0.01 compared with Sham/solid group, and at ^#^*p* < 0.05 and ^##^*p* < 0.01 compared with Sham/powder group. In panels **(F–H)** differences were considered significant at *p < 0.05 and **p < 0.01.

Mice were housed individually, and acclimated to a rearing environment for 1 week prior to the experiments and randomly assigned the Sham/solid, Sham/powder, or Malocclusion/powder group. Since mice with occlusal disharmony are unable to eat hard foods due to poor bite, a Malocclusion/solid group was not included. Random numbers were generated by Microsoft Excel. The mice in the powder diet-fed groups were acclimated to the powder diet for 3 days before surgery. The occlusal disharmony model of the anterior teeth simulates a stressful situation similar to that seen in humans and induces cognitive impairment (Yoshihara et al., 2009; Yoshihara and Yawaka, 2011; Shimizu et al., 2018; Suita et al., 2020). Occlusal disharmony was induced according to previously reported methods, with certain modifications (Shimizu et al., 2018; Suita et al., 2020). In brief, the mice were anesthetized by the intraperitoneal (ip) administration of a mixed anesthetic agent: 0.3 mg/kg medetomidine (Meiji Seika Pharma, Japan), 4.0 mg/kg midazolam (Sandoz, Tokyo, Japan), and 5.0 mg/kg butorphanol (Meiji Seika Pharma, Tokyo, Japan). Then, 1.0 mm of composite resin (BEAUTIFIL Flow Plus, SHOFU Inc., Kyoto, Japan) was applied to their mandibular anterior teeth with pre-treatment of FL-Bond II Primer and Bonding Agent (SHOFU Inc., Kyoto, Japan). Next, light curing was performed for 30 s at 1200 mW/cm^2^ in the vertical direction using a PEN Bright (SHOFU, Kyoto, Japan). The mice were recovered from anesthesia by the ip administration of 0.3 mg/kg atipamezole (Nippon Zenyaku Kogyo, Koriyama, Japan). The sham mice were anesthetized, underwent no intervention, and were recovered. All mice were individually housed for 1 month. Photographs and a schematic representation of the mouse occlusal disharmony model are shown in Figure 1A.

### 2.3. Food intake and body weight

Food intake and body weight of mice in three groups measured daily at 07:00. Body weight gain was calculated during each period. Food intake per body weight was calculated for each experimental day, and body weight gain per food intake between Days 1 and 4, Days 4 and 9, and Days 9 and 30.

### 2.4. Refeeding test

The mice used in the refeeding tests differed from those used in the behavior tests. The refeeding tests were performed according to the schedule diagram in Figure 1B. The mice in each group were fasted for 16 h and had *ad libitum* water access. Cumulative food intake was measured 0.5, 1, 2, and 4 h post-feeding.

### 2.5. NOR test

**FIGURE 2:**
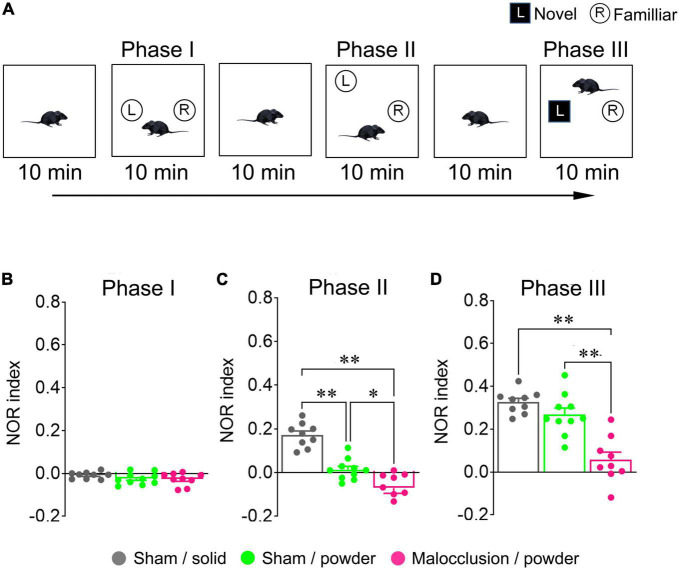
Schematic illustration of novel object recognition (NOR) test and NOR indices. All mice in Sham/solid (*n* = 9), Sham/powder (*n* = 10), and Malocclusion/powder group (*n* = 9) were subjected to NOR test on Day 28 **(A)**. NOR indices in Phases I **(B)**, II **(C)**, and III **(D)** were calculated. Data are represented as means ± SEM. Differences were considered significant at *p < 0.05 and **p < 0.01.

The NOR tests were performed according to a previous study (Rokot et al., 2021) and are shown in the schedule diagram in Figure 1B. Each mouse was placed in an empty 60 cm × 60 cm × 70 cm box with black walls and an open top for video recording. The mice were allowed to acclimate to the environment for 10 min (habituation phase) and returned to their home cages. Two objects of the same color, shape, and size were placed on opposite sides of each box. The mice were placed in the boxes, where they could freely explore for 10 min (Phase I), and returned to their home cages. The objects were removed from the boxes. The mice were placed in the cleaned, empty boxes for 10 min (resting phase) and returned to their home cages. The objects used in Phase I were replaced in the boxes. However, one item was placed in the same position as before (familiar), whereas the other was placed in a different position. The mice were placed in the boxes, allowed to explore freely for 10 min (Phase II), and returned to their home cages. The objects were removed from the boxes. The mice were then placed again in the cleaned, empty boxes for 10 min (resting phase). A familiar object and novel object of a different color, shape, and size were placed in the same positions as in Phase I. The mice were placed in the boxes and allowed to explore for 10 min (Phase III). All objects and the box were cleaned with 70% ethanol to remove any residual odors after each phase. Object exploration was defined as touching it with the nose but climbing onto it or chewing it was not considered exploration. In contrast, a mouse that sniffed the object and climbed was considered to have explored it. The NOR index was calculated as follows: (time of exploration of the new object–time of exploration of the familiar object)/(time of exploration of the new object + time of exploration of the familiar object). A schematic diagram of the NOR test procedure is shown in Figure 2A.

### 2.6. Tissue sampling

The mice were fasted for 4 h to reduce variability in the expression of their feeding-associated peptides. Tissues and peripheral blood were sampled, as described below, from the various mice subjected to the behavior test. The mice were anesthetized by the ip administration of a mixture of 0.3 mg/kg medetomidine, 4.0 mg/kg midazolam, and 5.0 mg/kg butorphanol. Peripheral blood was collected from the heart. Plasma was separated by centrifugation at 4°C and stored at −80°C until assay. The levels of blood glucose (BG), total cholesterol (T-CHO), and triglyceride (TG) in the plasma samples were measured in Kyudo Co., Ltd. (Saga, Japan). Mice were perfused with 0.1 M phosphate buffer and euthanized by perfusion without awakening from anesthesia. Brain tissues were excised and isolated for RT-qPCR. For immunohistochemistry analysis, the mice were perfused with 0.1 M phosphate buffer (pH 7.0) followed by 4% paraformaldehyde and 0.5% glutaraldehyde in 0.1 M phosphate buffer.

### 2.7. RT-qPCR

The mice were perfused with 0.1 M phosphate buffer and their hypothalami were isolated. Total RNA was extracted using the RNeasy Plus Mini Kit (No. 74134; QIAGEN, Hilden, Germany). The RNA was reverse transcribed to cDNA using SuperScript IV VILO (No. 11756050; Invitrogen, Carlsbad, CA, USA). RT-qPCR was performed using SYBR Green Master Mix (Thermo Fisher Scientific, Waltham, MA, USA) according to the manufacturer’s protocol. The primers used in RT-qPCR are listed in Supplementary Table 1.

### 2.8. Immunohistochemistry

Coronal sections (25 μm) of the hypothalami were cut on a cryostat (CryoStar NX70; Thermo Fisher Scientific, Waltham, MA, USA). Hypothalamus sections were incubated with rabbit anti-c-Fos antibody (1:100; ABE457; Merck Millipore, Belize, MA, USA) or mouse anti-c-Fos (1:500; sc-166940; Santa Cruz Biotechnology, Dallas, TX, USA), rabbit AgRP (1:1,000; H-003-53; Phoenix Pharmaceuticals, Burlingame, CA, USA), and guinea pig anti-product gene protein 9.5 (PGP9.5; 1:5,000; GP14104; Neuromics, Edina, MN, USA), which is a pan-neuronal marker (Schofield et al., 1995; Day and Thompson, 2010), at 4°C overnight. The sections were then incubated with the secondary antibodies Alexa Fluor 488-conjugated donkey anti-rabbit IgG (1:500; ab150065; Abcam, Cambridge, UK), Alexa Fluor 555-conjugated donkey anti-mouse IgG (1:500; ab150110, Abcam), and Alexa Fluor 647-conjugated donkey anti-guinea pig IgG (1:500; 706-605-148; Jackson ImmunoResearch Labs, West Grove, PA, USA) at 25°C for 3 h. The nuclei were stained with 4′,6-diamidino-2-phenylindole dihydrochloride solution (DAPI; No. D523; Dojindo Molecular Technologies Inc., Kumamoto, Japan). The image was observed using confocal laser microscopy (LSM 900; Carl Zeiss AG, Jena, Germany). The c-Fos-positive cells were enumerated on one side of each of six hypothalamus tissue sections per mouse at 200 × magnification using confocal laser microscopy. The averages were calculated and used in the subsequent analysis. The numbers of c-Fos- and DAPI-positive cells were manually counted, and c-Fos-positive cell numbers were normalized by the respective DAPI-positive cell numbers. AgRP and c- Fos-, DAPI and c- Fos-, AgRP and DAPI-, AgRP, and PGP9. 5-, and DAPI and PDP9.5-positive cells in arcuate nuclei were observed at 400 × magnification using confocal laser microscopy.

### 2.9. Cannula implantation

The mice were anesthetized by the ip administration of a mixture of 0.3 mg/kg medetomidine, 4.0 mg/kg midazolam, and 5.0 mg/kg butorphanol. A guide cannula (25-gauge; Eicom, Kyoto, Japan) was implanted into the right lateral ventricle with a Kopf stereotaxic frame (David Kopf Instruments, Tujunga, CA, USA). The stereotaxic coordinates were 0.8 mm posterior to the bregma, 1.5 mm left lateral to the midline, and 1.2 mm below the outer surface of the skull. The guide cannula was secured with dental cement (Super Bond; Sun Medical Co. Ltd., Moriyama, Japan) and anchored with two stainless steel screws (AN-3; Eicom) fixed to the dorsal surface of the skull. A dummy cannula (AD-4; Eicom) was placed into each guide cannula and fixed with a screw cap (AC-4; Eicom) to prevent occlusion. After the cannula implantation, the mice were recovered from anesthesia by the ip administration of 0.3 mg/kg atipamezole. The mice were lightly anesthetized by isoflurane inhalation and intracerebroventricular (icv) administration was performed. The dummy cannulae were replaced with microinjection cannulae (AMI-5; Eicom) that were 1 mm longer than the guide cannulae. Each microinjection cannula was connected to a polyethylene tube (PE-50; Clay Adams, Parsippany, NJ, USA). At the end of the experiments, the mice were euthanized by carbon dioxide inhalation and the correct locations of the icv cannulae were verified with 10 μL of 0.05% cresyl violet dye.

### 2.10. Drug administration

Anti-AgRP antibody (AF634; R&D Systems, Minneapolis, MN, USA) was dissolved in saline solution (0.9% NaCl) and administered intracerebroventricularly at a dose of 0.1 μg/mouse from Day 25 to 30 at 07:00-08:00. Saline solution (2 μL) was also intracerebroventricularly administered as a vehicle. Specificity of this antibody was verified in previous studies (Cortes-Campos et al., 2013; Okamoto et al., 2016; Fukuhara et al., 2019; Ou et al., 2019; Kim et al., 2020).

### 2.11. Data analysis

Data are represented as means ± standard error of the mean (SEM). Pairwise comparisons between groups were conducted using Student’s *t*-test. One-way ANOVA followed by Tukey’s multiple comparisons tests was used to compare the three groups. Two-way ANOVA followed by Tukey’s or Bonferroni’s multiple comparisons tests were used to compare two or three groups over time. Pearson correlation coefficient was used to explore the correlations between variables the variables. Differences were considered statistically significant at *p* < 0.05. All statistical analyses were performed using GraphPad Prism 9 (GraphPad Software, La Jolla, CA, USA).

## 3. Results

### 3.1. Mice with malocclusion had lower body weight but no alteration in food intake

The mean body weight of the Malocclusion/powder group was significantly lower than that of the Sham/solid group between Days 1 and 30 and Sham/powder group between Days 1 and 12 (*F*_2,25_ = 9.014, *p* = 0.0011, two-way ANOVA; Figure 1C). The mean body weight of the Sham/powder group was significantly lower than that of the Sham/solid group on Day 26 (Figure 1C). Food intake was significantly lower in the Malocclusion/powder group than in the Sham/solid and Sham/powder groups on Days 1 and 2 (*F*_2,25_ = 6.166, *p* = 0.0065, two-way ANOVA; Figure 1D). Since the mean body weight of the Malocclusion/powder group was significantly lower than that of the Sham/solid group, food intake/body weight and body weight gain/food intake was calculated to further assess the effects of malocclusion. The mean of food intake/body weight in the Malocclusion/powder group was lower between Days 1 and 2 but higher between Days 4 and 7 than that in the other groups and higher than that in the Sham/powder group on Day 8 (Figure 1E). However, there were no significant differences observed among the groups between Days 9 and 30 (*F*_2,25_ = 1.382, *p* = 0.2697, two-way ANOVA; Figure 1E). Moreover, the mean of body weight gain/food intake in the Malocclusion/powder group was significantly lower than that of the Sham/solid group on Days 1, 21, and 26 as well as that of the Sham/powder group on Days 1, 2, and 21 (*F*_2,25_ = 16.02, *p* < 0.001, two-way ANOVA; Figure 1E). Furthermore, the body weight gain/food intake was significantly lower in the Malocclusion/powder group than that in the other groups from Day 1 to 4 (*F*_2,25_ = 15.47, *p* < 0.0001, one-way ANOVA; Figure 1F). Finally, the BG and TG concentrations in peripheral blood showed no differences among the groups (BG: *F*_2,23_ = 1.165, *p* = 0.3296, TG: *F*_2,23_ = 0.6130, *p* = 0.5503, one-way ANOVA; Figure 1G). Notably, the T-CHO concentration in the peripheral blood of the Sham/solid group was significantly higher than that of the other groups (*F*_2,23_ = 9.645, *p* = 0.0009, one-way ANOVA; Figure 1G).

### 3.2. Malocclusion increased cumulative post-fasting food intake in mice

In the refeeding test, the cumulative food intake was significantly higher in the Malocclusion/powder group than in the Sham/solid and Sham/powder groups after 1 and 2 h (*F*_2,25_ = 3.692, *p* = 0.00394, two-way ANOVA; Figure 1H). Nevertheless, there were no significant differences in the cumulative food intake among groups after 4 h (Figure 1H).

### 3.3. Malocclusion impaired cognitive function in NOR test

There were no significant differences in Phase I NOR indices among groups (*F*_2,25_ = 1.420, *p* = 0.26, one-way ANOVA; Figure 2B). However, the Phase II NOR index was lower in the Malocclusion/powder group than in the Sham/solid and Sham/powder groups, and the Phase II NOR index was lower in the Sham/powder than in the Sham/solid group (*F*_2,25_ = 39.53, *p* < 0.001, one-way ANOVA; Figure 2C). The Phase III NOR index was significantly lower in the Malocclusion/powder group than in the Sham/solid or Sham/powder groups, and the Phase III NOR index of the Sham/powder group reverted to the level of that of the Sham/solid group (*F*_2,25_ = 23.41, *p* < 0.001, two-way ANOVA; Figure 2D).

### 3.4. Hypothalamic mRNA levels of AgRP and Ucn2 were increased in mice with malocclusion

**FIGURE 3:**
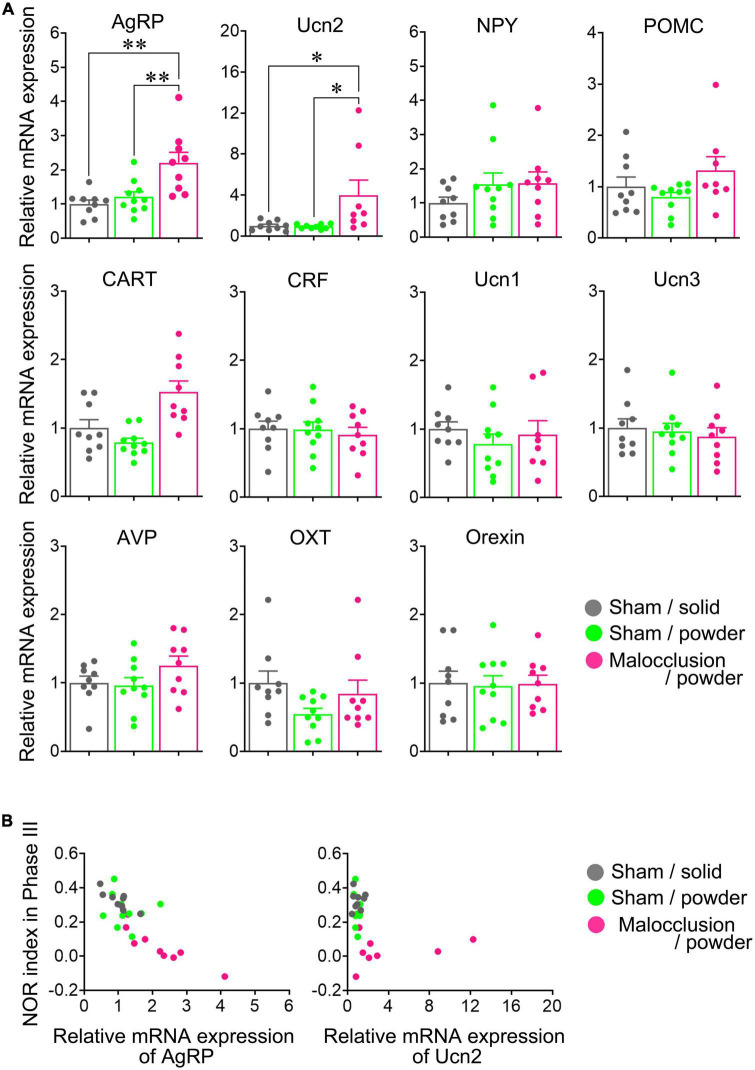
Hypothalamic neuropeptide mRNA levels. **(A)** mRNA levels of AgRP, agouti-related peptide; NPY, neuropeptide Y; POMC, proopiomelanocortin; CART, cocaine- and amphetamine-regulated transcript; CRF, corticotropin-releasing factor; Ucn, urocortin 1, Ucn2, Ucn3; AVP, arginine vasopressin; OXT, oxytocin, and orexin were measured in hypothalami isolated on Day 30. **(B)** Correlation between mRNA expressions of AgRP or Ucn2 and Phase III NOR indices are represented with respective Pearson correlation coefficients. Data are represented as means ± SEM (*n* = 8–10). Differences were considered significant at *p < 0.05 and **p < 0.01.

The AgRP and urocortin2 (Ucn2) mRNA levels were significantly higher in the Malocclusion/powder group than in the Sham/solid and Sham/powder groups (AgRP: *F*_2,25_ = 9.574, *p* < 0.001, Ucn2: *F*_2,23_ = 4.553, *p* = 0.0216, one-way ANOVA; Figure 3A). In contrast, the mRNA levels of NPY, POMC (proopiomelanocortin), CART (cocaine- and amphetamine-regulated transcript), CRF (corticotropin-releasing factor), Ucn1, Ucn3, AVP (arginine vasopressin), OXT (oxytocin), and orexin did not significantly differ among groups (Figure 3A). Interestingly, although the Phase III NOR indices were negatively correlated with mRNA expression levels of AgRP (*r* = −0.8166, *p* < 0.01, Figure 3B), there was no correlation between Phase III NOR indices and mRNA expression levels of Ucn2 (Figure 3B).

### 3.5. Number of c-Fos-positive cells in the arcuate nucleus of the hypothalamus was increased in mice with malocclusion, and these cells were AgRP-positive

**FIGURE 4:**
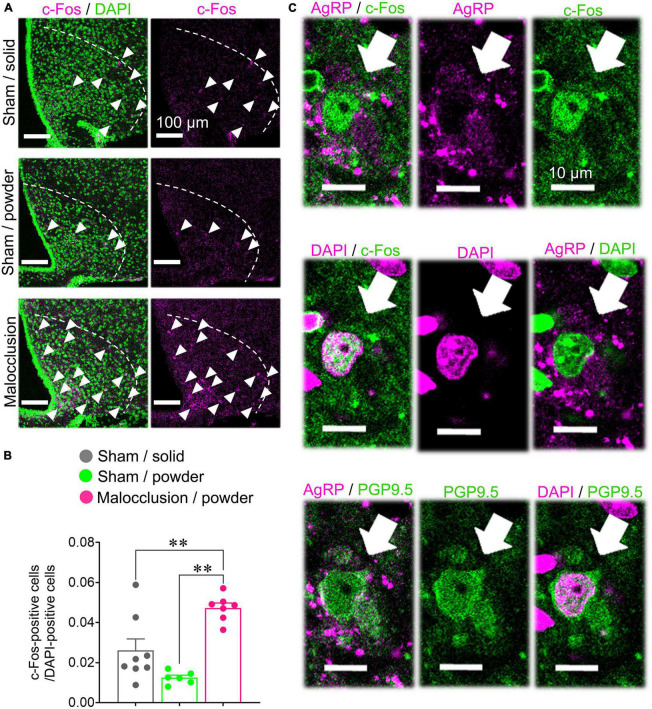
Immunostaining for c-Fos and AgRP. Coronal sections of arcuate nuclei were stained with anti-mouse c-Fos and/or anti-AgRP antibody. **(A)** Representative images of c-Fos-positive cells (white arrowheads) in arcuate nucleus (white dashed line) when subjected to immunofluorescent staining. **(B)** c-Fos-positive cells were enumerated on one side of each of the six hypothalamus tissue sections per mouse in Sham/solid (*n* = 8), Sham/powder (*n* = 6), and Malocclusion/powder group (*n* = 7). The numbers of c-Fos-positive cells were normalized by DAPI-positive cells. Data are represented as means ± SEM. Differences were considered significant at **p < 0.01. **(C)** Representative images of AgRP- and c- Fos-, DAPI- and c- Fos-, AgRP- and DAPI-, AgRP- and PGP9. 5-, and DAPI- and PGP9.5-positive cells in arcuate nuclei subjected to immunofluorescent staining (white arrows).

We examined the arcuate nuclei of the hypothalami as the AgRP neurons are localized there. There were significantly more c-Fos-positive cells in the arcuate nuclei of the Malocclusion/powder group than in those of the Sham/solid and Sham/powder groups (*F*_2,18_ = 16.68, *p* < 0.001, one-way ANOVA; Figures 4A, B). Moreover, the c-Fos-positive cells were AgRP-positive (Figure 4C).

### 3.6. Icv anti-AgRP antibody administration reversed malocclusion-induced cognitive impairment

**FIGURE 5:**
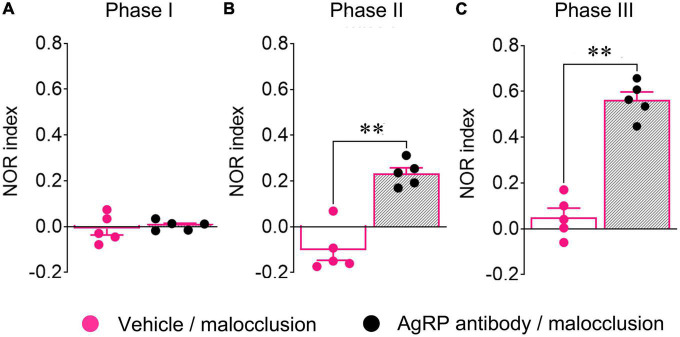
Novel object recognition (NOR) indices of mice subjected to intracerebroventricular (icv) anti-AgRP antibody administration. Anti-AgRP antibody (0.1 μg/2 μL/mouse) was administered intracerebroventricularly to all mice from Day 25 to 30. NOR tests were performed 30 min after icv administration on Day 30. Phase I **(A)**, Phase II **(B)**, and Phase III **(C)** NOR indices of Malocclusion/powder group mice administered vehicle (*n* = 5) or antibody (*n* = 5). Data are represented as means ± SEM. Differences were considered significant at **p < 0.01.

A previous study demonstrated that Ucn2 has no effect on NOR other than influencing the number of attempts to climb the new object (Clark et al., 2007), and the present study revealed that mRNA expression of AgRP, not Ucn2, had a negative correlation with NOR indices in Phase III (Figure 3B). For these reasons, we focused on AgRP alone. The Phase I NOR indices did not differ between mice administered antibody and those administered vehicle [t(8) = 0.4876, *p* = 0.6389, *t*-test; Figure 5A]. The Phase II and Phase III NOR indices were significantly reversed in the Malocclusion/powder group administered anti-AgRP antibody by icv for 5 days [t(8) = 6.534, *p* = 0.0002; Figure 5B and t(8) = 9.629, *p* < 0.0001, *t*-test; Figure 5C]. Icv anti-AgRP antibody administration did not alter body weight or food intake relative to vehicle administration (*F*_1,8_ = 1.394, *p* = 0.27 and *F*_1,8_ = 3.493, *p* = 0.10, two-way ANOVA; Supplementary Figure 1).

## 4. Discussion

The mean body weight of the Malocclusion/powder group was significantly lower than that of the Sham/solid group for 30 days and significantly lower than that of the Sham/powder group for the first 11 days. Food intake was significantly lower in the Malocclusion/powder group than in the other groups for the first 2 days. While the food intake/body weight of the Malocclusion/powder group was lower for the first 2 days after the induction of occlusal disharmony, it became higher than that of other groups between Days 4 and 8, returning to normal levels from then on. Moreover, the body weight gain/food intake between Days 1 and 4 was lower in the Malocclusion/powder group than in the other groups. However, this value returned to normal levels between Days 4 and 30. The total cholesterol concentrations in the peripheral blood samples from both powder diet-fed groups were lower than those from the Sham/solid group. However, a decline in the Phase III NOR indices was observed in the Malocclusion/powder group, yet not in the Sham/powder group. Additionally, the decrease in cholesterol showed no effect on body weight gain. A previous study demonstrated that the high cholesterol level seen in an obesity model induced by the Cafeteria diet impairs cognitive function in the NOR task (Lewis et al., 2019). Hence, the resin construction surgery itself had virtually no influence as of the day upon which the behavior tests and tissue samplings were conducted. BG levels in all groups were slightly increased by the anesthesia (Ochiai et al., 2016). Occlusal disharmony induced by the composite resin applied on the mandibular incisors reduces body weight (Suita et al., 2020). The present study showed lower body weight in the Malocclusion/powder group than in the Sham/solid group. This finding was consistent with previous studies.

In present study both Sham/powder presented with decreased the NOR index in Phase II of NOR test. On the other hand, Malocclusion/powder group presented with decreased both NOR indices in Phase II and III. Occlusal disharmony may lead to abnormal sensory input and is associated with working memory function impairment (Sakatani et al., 2013). The NOR task is considered to evaluate the involvement of working memory in object location and features, memory consolidation, and reorganization of consolidated memory associated with input of new information (Antunes and Biala, 2012). One of key areas of the brain involved in this processing is the hippocampus (Furini et al., 2020). In a previous study, occlusal disharmony impaired cognitive ability of young mice when performing the NOR task 1 and 4 weeks after the loading (Maeshiba et al., 2022). Moreover, the protein levels of various cognitive suppressor molecules in the hippocampus, such as amyloid-β and phosphorylated tau, were increased at 1 week and were reduced at 4 weeks after the loading (Maeshiba et al., 2022). Anorexigenic peptide signals in the hypothalamus have been reportedly associated with anorexia-induced recognition decline in juvenile mice (Rokot et al., 2021). Therefore, the hypothalamus may also be involved in malocclusion-induced cognitive decline in young mice.

Previous study reported that mice fed a powdered diet have lower position recognition test scores than those fed a solid diet and decrease in hippocampal nerve growth-promoting factor BDNF levels (Fukushima-Nakayama et al., 2017). Although Malocclusion/powder group presented the positional recognition decline in the present study, the decline was reversed by central administration of ant-AgRP antibody. The decline in positional recognition induced by malocclusion may have a different mechanism from that induced by a powder diet.

Hypothalamic AgRP mRNA was significantly upregulated in the Malocclusion/powder group. AgRP neuron activation occurred in response to malocclusion and was suppressed by central AgRP antibody administration. Upregulated hypothalamic AgRP is associated with significantly lower NOR test scores in mouse anorexia models (Rokot et al., 2021). Likewise, while the AgRP signal produces an aversive condition, its inhibition enhances the learning of a sensory cue-initiated food-acquisition task (Berrios et al., 2021). By blocking AgRP activity via icv antibody administration, the recognition ability of the Malocclusion/powder group may increase compared to that of Sham/solid mice. Chronic, unpredictable, mild stress impaired the recognition ability of rats in the NOR task, and the melanocortin four receptor, a receptor to which AgRP binds, was upregulated in the nucleus accumbens (Goudarzi et al., 2020). AgRP neurons project to the ventral striatum, including the nucleus accumbens, and contribute to motivation induced by dopamine signals (Reichenbach et al., 2022). The nucleus accumbens is an important area not only associated with the reward system, but also recognition memory such as taste neophobia (Alejandro Borja et al., 2020). Thus, the AgRP signal may be a key mediator of malocclusion-induced cognitive decline.

AgRP is a potent cerebral orexigenic peptide (Sohn, 2015). The Malocclusion/powder group exhibited no alteration in daily food intake between Day 4 and 30. Nevertheless, the cumulative food intake after overnight fasting increased until 2 h and normalized after 4 h compared with the other groups. AgRP and NPY contribute to food intake during 3 h of refeeding after 8 h of fasting (Palou et al., 2009). The observed increase in food intake in the Malocclusion/powder group at 2 h may reflect significant AgRP neuron activation.

A prior survey disclosed a negative correlation between malocclusion severity (assessed by the Index of Orthodontic Treatment Need-Dental Health Component) and mastication (assessed by food intake ability) (Choi et al., 2016). The association between eating disorders and occlusal disharmony has been shown (Chiba et al., 2022), and individuals with anorexia display perturbation of cognitive function (Kaye, 2008) and elevated plasma AgRP levels (Moriya et al., 2006). Patients with anorexia do not eat even when AgRP is activated (Escelsior et al., 2022). Suppression of AgRP signaling reverses the decline in cognitive function in a mouse anorexia model (Rokot et al., 2021). In the present study, the Malocclusion/powder group exhibited no alteration in daily food intake even though their AgRP neurons were activated. Thus, there may be certain neurophysiological similarities between malocclusion and anorexia. AgRP signaling can be a new therapeutic target for cognitive decline in occlusal disharmony and anorexia nervosa.

The present study also showed that Ucn2 mRNA expression was upregulated in the Malocclusion/powder group. Ucn2 is a CRF family peptide that suppresses food intake and gastric motility and is anxiolytic (Martínez et al., 2004). Central Ucn2 infusion alters the frequency with which animals climb onto new objects but has no apparent effect on latency to touch the new object or the total number of touches and climbs onto the new object (Clark et al., 2007). Hence, Ucn2 may have little effect on cognitive function. For this reason, we focused on assessing the effects of AgRP in the present study. The anorexigenic effect of Ucn2 may have contributed to the observed lack of change in food intake in the Malocclusion/powder group despite the increase in levels of the orexigenic peptide AgRP. Although AgRP neurons in the hypothalamus contribute to CRF activity in a fasted state (Fernandes et al., 2022), the interaction between AgRP and Ucn2 has not yet been reported. The cognitive impairment in NOR test correlated with mRNA expression of AgRP, not Ucn2, in the present study. In summary, Ucn2 may act independently of AgRP in mice with malocclusion.

Corticotropin-releasing factor is a peptide that regulates various stress responses that affect gastrointestinal function and induce anxiety and the secretion of stress-related hormones, such as corticosterone in rodents and cortisol, in humans (Deussing and Chen, 2018). Chronic stress may induce other factors besides, or in addition to, CRF (Ataka et al., 2012). Chronic homotypic (but not heterotypic) stress is not associated with any alteration in hypothalamic CRF (Zheng et al., 2010). Although malocclusion is a stressor, no hypothalamic CRF mRNA upregulation was observed in the Malocclusion/powder group here. Malocclusion may be a form of chronic homotypic stress. Plasma corticosterone levels do not change for 4 weeks in a rat malocclusion model (Irie et al., 2011). The duration of malocclusion was 4 weeks in the present study. CRF may not have been implicated in the mechanism of malocclusion-induced cognitive impairment.

The present work had certain limitations as we did not perform gene silencing to remove the target mRNA nor did we use knockout mice. Additionally, we did not identify the projection target of AgRP. Further research is needed to more thoroughly explore the complex interaction malocclusion-induced cognitive decline and AgRP signaling. Additionally, the results of this study should be corroborated or validated in future clinical studies. However, the present study is the first to show that AgRP signaling in the arcuate nucleus of the hypothalamus contributes to occlusal disharmony-induced recognition decline. AgRP signaling might be a novel target for the treatment of a defect of cognitive ability induced by the occlusal disharmony.

## Data availability statement

The original contributions presented in this study are included in the article/Supplementary material, further inquiries can be directed to the corresponding author.

## Ethics statement

This animal study was reviewed and approved by the Kagoshima University Committee for Animal Experiments (No. D21035).

## Author contributions

JK contributed to the study conception, design, data acquisition, interpretation, analysis, drafted, and critically revised the manuscript. KA and HI contributed to the study design, data acquisition, interpretation, analysis, drafted, and critically revised the manuscript. YO contributed to the study conception, design, data interpretation, drafted, and critically revised the manuscript. KY, KM, and TI contributed to the data interpretation, drafted, and critically revised the manuscript. AA contributed to the study design, data interpretation, and critically revised the manuscript. SM contributed to the study conception, design, data interpretation, and critically revised the manuscript. All authors approved the final version of the manuscript and agreed to be accountable for all aspects of the work.
