# Supplementary material for: Malocclusion impairs cognitive behavior via AgRP signaling in adolescent mice
Source: Front Neurosci. 2023 Apr 24;17:1156523. doi: 10.3389/fnins.2023.1156523 (PMC10164942; doi:10.3389/fnins.2023.1156523)
Supplement: Supplementary file 1 [file Data_Sheet_1.docx]

Supplementary Material

Malocclusion Impairs Cognitive Behavior via AgRP Signaling in Adolescent Mice

**Junya Kusumoto^*^, Koji Ataka^*^, Haruki Iwai^*^, Yasuhiko Oga, KeitaYamagata, Kanako Marutani, Takanori Ishikawa, Akihiro Asakawa, Shoichi Miyawaki**

*** Correspondence:** Dr. Koji Ataka: [kataka@kobepharma-u.ac.jp](mailto:kataka@kobepharma-u.ac.jp)

# Supplementary Methods

## Animals

In the present study, n refers to the number of animals. Each mouse was considered to be an experimental unit within this study. We set the humane endpoint as follows. If abnormal behavior, such as immobility or tremor, was observed during the experiment, animals were euthanized with an overdose of anesthesia. Abnormal mice were not observed in the present study. Although 92 mice were used in this study, the resin of 3 mice and icv cannulae of 2 mice became dislodged during the operation. Therefore, 87 mice were included in this study. The number of mice in each experiment was as follows. We used the same mice in body weight and food intake (Fig. 1C-E), measurements of glucose, total cholesterol, and triglyseride in perioheral blood, NOR behavior (Fig. 2B-D), and hypothalamic mRNA level (Fig. 3) experiments. These experiments started with 29 mice (Sham/solid group: 9, Sham/solid powder: 10, and Malocclusion/powder group: 10) but 1 mouse in the Malocclusion/powder group was excluded from the analysis because the resin fell off in the middle of the experiment. And more, 1 mouse in the Sham/powder and 1 mouse in the Malocclusion/powder group were excluded from the blood analysis because sampling was fault. all The cumulative post-fasting food intake experiment started with 30 mice (Sham/solid group: 10, Sham/powder group: 10, and Malocclusion/powder group: 10) but 2 mice in the Malocclusion/powder group were excluded from the analysis because their resins fell off in the middle of the experiment (Fig. 1F). We randomly selected 21 mice (Sham/solid group: 8, Sham/powder group: 6, and Malocclusion/powder group: 7) from mice used in the cumulative post-fasting food intake experiment for immunostaining (Fig. 4). The icv anti-AgRP antibody administration experiment started with 12 mice, but 2 mice were excluded from the analysis because their ICV cannulae fell off in the middle of the experiment. Finally, each of the five mice treated with vehicle and AgRP antibody was examined using the NOR test (Fig. 5).

# Supplementary Figures and Tables

## Supplementary Tables

Table S1. Primers used in RT-qPCR

Forward Reverse

neuropeptide Y (NPY) NM_023456 CGCTCTGCGACACTACATCAAT TGAGATGAGGGTGGAAACTTGG

agouti-related peptide (AgRP) NM_007427 GGACTGAGCATAAAGATGGCATGA TGTAGCCAGGGCATGAGGTG

proopiomelanocortin (POMC) NM_001278581 AATTACGTGGGTTATAGGACAGGAC CCCTGAGCGACTGTAGCAGA

cocaine- and amphetamine-regulated transcript (CART) NM_013732 GACATCTACTCTGCCGTGGATGA TTCTTGCAACGCTTCGATCTG

corticotropin-releasing factor (CRF) NM_205769 CAGAGCCCAAGTACGTTGAGAG GCTCTCTTCTCCTCCCTTGGTA

urocortin1 (Ucn1) NM_021290 CATCTTGCACTGGGCAGACACT AAGCTGTGCCAAGAGCAGCAAC

urocortin2 (Ucn2) NM_145077 GACAGCCACAAAGCTGGACAGTA GGCTCAGAAGCATGGCAAGA

urocortin3 (Ucn3) NM_031250 CCACTCCAGAGCAAAGTCCACTTAC GCTCAGCAAGGGCACATCTTC

arginine vasopressin (AVP) NM_009732 TCTCTGACATGGAGCTGAGACAG AGGGCAGGTAGTTCTCCTCCT

oxytocin (OXT) NM_012996 TGCCAGGAGGAGAACTACCTG TATTCCCAGAAAGTGGGCTCAG

orexin NM_010410 CGTAACTACCACCGCTTTAGCA TGCCATTTACCAAGAGACTGACA

glyceraldehyde-3-phosphate dehydrogenase (GAPDH) NM_008084 TGTGTCCGTCGTGGATCTGA TTGCTGTTGAAGTCGCAGGAG

## Supplementary Figures


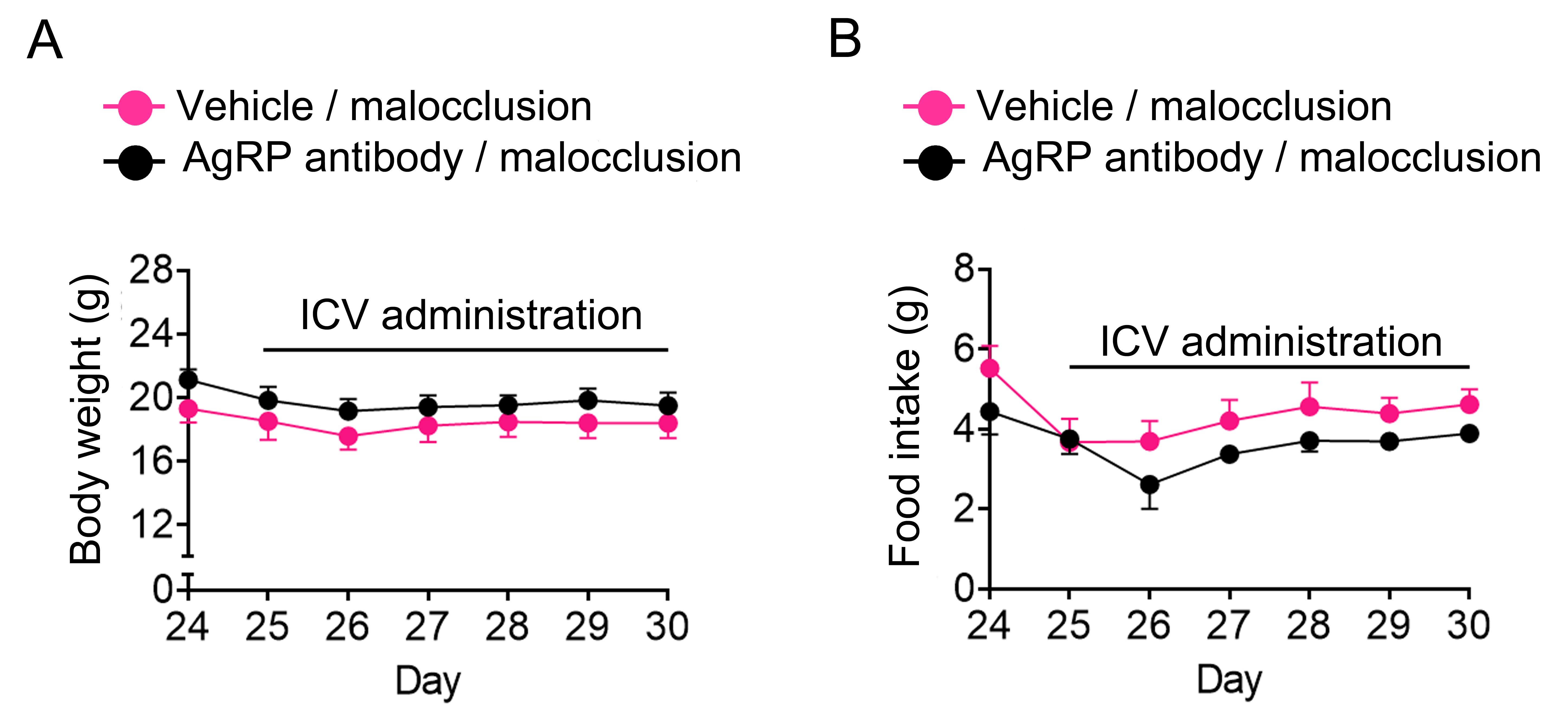


Figure S1. Time courses of body weight and food intake in mice repeatedly subjected to intracerebroventricular (icv) anti-AgRP antibody administration. (A, B) Body weight (A) and food intake (B) of mice in the Malocclusion/powder group after icv administration of vehicle (*n* = 5) or anti-AgRP antibody (*n* = 5). Data are shown as means ± SEM. Bar indicates icv administration period.
